# Successful identification of rare variants using oligogenic segregation analysis as a prioritizing tool for whole-exome sequencing studies

**DOI:** 10.1186/1753-6561-5-S9-S11

**Published:** 2011-11-29

**Authors:** France Gagnon, Nicole M Roslin, Mathieu Lemire

**Affiliations:** 1 Dalla Lana School of Public Health, University of Toronto, 155 College Street, Toronto, ON M5T 3M7, Canada; 2 Hospital for Sick Children Research Institute, 555 University Avenue, Toronto, ON M5G 1X8, Canada; 3 Ontario Institute for Cancer Research, 101 College Street, Suite 800, Toronto, ON M5G 0A3, Canada

## Abstract

We aim to identify rare variants that have large effects on trait variance using a cost-efficient strategy. We use an oligogenic segregation analysis as a prioritizing tool for whole-exome sequencing studies to identify families more likely to harbor rare variants, by estimating the mean number of quantitative trait loci (QTLs) in each family. We hypothesize that families with additional QTLs, relative to the other families, are more likely to segregate functional rare variants. We test the association of rare variants with the traits only in regions where at least modest evidence of linkage with the trait is observed, thereby reducing the number of tests performed. We found that family 7 harbored an estimated two, one, and zero additional QTLs for traits Q1, Q2, and Q4, respectively. Two rare variants (C4S4935 and C6S2981) segregating in family 7 were associated with Q1 and explained a substantial proportion of the observed linkage signal. These rare variants have 31 and 22 carriers, respectively, in the 128-member family and entered through a single but different founder. For Q2, we found one rare variant unique to family 7 that showed small effect and weak evidence of association; this was a false positive. These results are a proof of principle that prioritizing the sequencing of carefully selected extended families is a simple and cost-efficient design strategy for sequencing studies aiming at identifying functional rare variants.

## Background

Genome-wide association scans (GWAS) have been successful at identifying common variants associated with common diseases or quantitative traits. GWAS have benefited from international efforts to catalog a substantial proportion of the common variants (generally thought to be those with allele frequency above 5%) found in the genome and characterize the linkage disequilibrium structure between them [1]. Because the designs of GWAS are based on genotyping only a carefully selected set of common single-nucleotide polymorphisms (SNPs) that are at most loosely correlated to one another (a set of tagging SNPs), it is still difficult to infer causality from the observed associations. Large follow-up resequencing efforts are necessary to attempt to locate the functional variants that can explain the associations identified by GWAS. True functional variants might have been only indirectly detected by GWAS through linkage disequilibrium; although these functional variants are likely to be in the uncommon to common frequency range, the possibility exists that some associations identified by GWAS are truly caused by rare variants (generally thought to be those with allele frequency less than 1%) that have a large effect on the disease or the trait [2]. Because only a few individuals are expected to be carriers of a rare allele, methods that are based on the accumulation of rare alleles across a set of rare SNPs have been developed for samples of unrelated individuals [3,4]. For instance, the proportion of case and control subjects who are carriers of at least one rare allele at any one of the SNPs in the set can be compared and their combined effect tested. If all SNPs in the set are truly functional, then these accumulation methods benefit from the increase in the effective frequency of the set [5]. But because the set of SNPs is also likely to include SNPs that are truly nonfunctional, perhaps even in larger numbers, the difficulty in inferring causality remains.

We present a family-based study design strategy to help identify specific rare variants, potentially functional, that are associated with a genetically determined quantitative trait. The strategy relies on first performing an oligogenic segregation analysis of the trait in a sample of extended families, analyzing each family individually. This analysis allows us to identify the families which are more likely to harbor rare variants that explain a significant proportion of the trait variance, that is, families who carry more quantitative trait loci (QTLs). Rare alleles are unlikely to segregate in more than a few families, especially if families are not ascertained on the basis of the presence of a disease or extreme trait values but rather are population-based samples, as is the case for the simulated Genetic Analysis Workshop 17 (GAW17) family data. Once a rare allele enters a family, the allele can segregate to many more family members, making extended-family designs a natural choice for the identification of specific rare functional variants. Identifying and prioritizing families which are more likely to harbor rare QTLs can reduce the multiple testing burden associated with testing a (potentially large) number of rare, mostly nonfunctional variants spread over many families as well as reduce the sequencing cost.

## Methods

For each quantitative trait, we estimate the mean number of QTLs explaining a proportion of the variance of the trait and their individual effect sizes, assuming an oligogenic linear model. In this model, the trait *Y* is modeled as:(1)

where *μ* is the overall mean, *X* is the design matrix of covariates, *β* is the vector of covariate effects, *Q_i_* is the design matrix of the additive and dominant components of the *i*th QTL, *α_i_* is the vector of the *i*th QTL effect, and *e* is the normally distributed residuals. Using the reversible jump Markov chain Monte Carlo algorithm implemented in the program Loki [6], the number of QTLs *k* is allowed to be an estimable parameter of the model. The number of QTLs that each family is likely to harbor can be estimated by analyzing each family separately. Sex, Age, and Smoking status of each participant are included as covariates.

Families who are estimated to harbor at least one additional QTL, compared to the average number of QTLs in the other families, are tested for linkage at each one of the genes where fully informative identity-by-descent sharing is available. We hypothesize that these additional QTLs are caused by rare functional variants (whose effects are detectable) that are segregating in that family. We use a variance components oligogenic linkage approach, implemented in the software SOLAR [7], to evaluate the evidence of linkage with the quantitative traits, keeping Sex, Age, and Smoking status of the individuals as covariates whenever they are declared significant predictors at *p* < 0.10. Regions surrounding a LOD of 0.60 (corresponding to a pointwise *p*-value of 0.05) are further investigated for the presence of rare functional variants. Within a 1-Mbp window centered at the position where the family-specific LOD score is detected, we extract all variants that enter that family through at most two founders. If at least five copies of a rare allele are seen in the family, the variant is included as a covariate in the model, in order to test its association with the trait and to determine how much of the linkage signal can be explained by the variant.

These analyses are performed using replicate 1 of the simulated GAW17 phenotypes, without knowledge of the underlying simulated model.

## Results

Table 1 shows the results of the oligogenic segregation analysis performed on the three quantitative traits. The proportions of the total variance of the traits attributable to each covariate and the proportion of the variance attributed to all QTLs are indicated as well as the expected number of QTLs that are likely to be segregating in all families. The three quantitative traits appeared to be genetically determined, with heritabilities of about 54% (Q1), 42% (Q2), and 64% (Q4) (heritability is defined as the ratio of the variance attributed to the QTLs to the variance not explained by the covariates).

**Table 1:** Results of the segregation analyses of the three available quantitative traits

Trait	Sex (%)	Age (%)	Smoking status (%)	QTL (%) (number of QTLs)	Residual (%)	Heritability (%)
Q1	0.3	10.3	4.1	46.5 (4.7)	38.9	54.4
Q2	0.3	0.3	0.2	41.8 (3.7)	57.4	42.1
Q4	1.4	73.8	3.3	13.8 (3.5)	7.6	64.5

Sex, Age, and Smoking status of the participants were included as covariates. Indicated are the percentages of the variance of the traits that are explained by each covariate, all QTLs taken together (along with the expected number of QTLs), and the residuals. Heritability is defined here as the ratio of the variance attributed to the QTLs (column 5) to the variance not explained by the covariates (column 5 + column 6).

Table 2 shows the number of QTLs that are likely to be segregating in each one of the eight extended families that formed the sample. For trait Q1, family 7 (consisting of 128 members, including 37 founders) was estimated to harbor two more QTLs than the number expected to be found in the other families. For trait Q2, the same family was expected to harbor one additional locus. For trait Q4, no family was estimated to have at least one more QTL than the other families, and so this trait was not analyzed further.

**Table 2:** Number of QTLs that are expected to be found in each family

Family	Family size	Q1	Q2	Q4
1	86	1.7	1.0	1.8
2	100	1.5	1.3	1.7
3	90	1.3	1.0	1.6
4	74	1.7	2.1	1.9
5	73	1.9	1.0	2.6
6	73	1.2	1.1	1.0
7	128	3.6 (+ 2.1)	2.24 (+ 1.0)	2.3
8	73	1.1	1.1	1.4

Whenever a family is expected to harbor at least one additional QTL than the other families, on average, the number of additional QTLs is indicated in parentheses.

We hypothesize that the additional loci in Q1 and Q2 reflect the presence of rare alleles that are segregating mostly, if not uniquely, in family 7. To help identify these rare functional alleles, we investigated regions of the genome where only that family showed at least some modest evidence of linkage with the trait (LOD of at least 0.6, corresponding to a *p*-value of 0.05). For trait Q1, 505 genes were in regions that attained LOD > 0.6; we observed a maximum LOD of 5.3 (*p* = 4 × 10^–7^) for *TLL1* and *ANP32C*, both located on chromosome band 4q32.3. For trait Q2, 315 genes attained a LOD > 0.6, with a maximum LOD of 2.02 (*p* = 0.001) at *UBASH3A* and *SLC37A1* (21q22.3). The regions surrounding all these genes (± 500 kb) included 216 variants for trait Q1 and 85 variants for trait Q2; these variants entered family 7 through at most two founders, and at least five copies of the rare alleles were seen among all the family’s members.

Each of these variants was tested for association with the trait in family 7, and we evaluated how much of the linkage the variants explained. Table 3 shows the variants that were significantly associated with a trait, even after accounting for the number of SNPs tested for that trait (i.e., after a Bonferroni correction of 216 tests for Q1 and 85 tests for Q2). Some of the SNPs in Table 3 were found to be in high linkage disequilibrium with each other in family 7; for the SNP pairs C5S12-C5S252 and C21S1096-C21S898 and the SNP triplet C11S2779-C11S2804-C11S3874, carriers of the rare allele at one SNP were also carriers of the rare allele at another SNP. Moreover, all but one carrier of the rare allele at C6S2432 were also carriers of the rare allele at C6S2981. To disentangle the associations seen at these SNPs and to refine the role of each, we evaluated their effects in the other families.

**Table 3:** Tests of association between variants and traits Q1 and Q2

Trait	SNP (Gene)	Family	Number of copies (number in founders)	Association *p*-value	Unadjusted LOD	Adjusted LOD	Variation due to SNP (%)
Q1	**C4S4935 (*VEGFC*)**	7	31 (1)	7.87 × 10^–12^	4.96	0.00	33.11
		Others	0 (0)	NA	NA	NA	NA
		All	31 (1)	1.12 × 10^–16^	5.12	0.00	10.64
	**C6S2981 (*VEGFA*)**	7	22 (1)	1.25 × 10^–7^	2.80	0.03	28.90
		Others	24 (2)	1.27 × 10^–8^	1.28	0.00	4.97
		All	46 (3)	9.90 × 10^–18^	4.91	0.00	11.96
	C6S2432 (*PSMB8*)	7	23 (1)	7.78 × 10^–7^	2.37	0.03	25.18
		Others	0 (0)	NA	NA	NA	NA
		All	23 (1)	3.95 × 10^–11^	4.58	0.94	8.94
	C6S5169 (*MCM9*)	7	21 (2)	3.37 × 10^–5^	1.91	0.04	17.34
		Others	15 (4)	0.90	0.08	0.08	0.00
		All	36 (6)	4.39 × 10^–5^	1.60	0.34	3.05
	C11S2779 (*OR10W1*)	7	16 (1)	1.64 × 10^–4^	0.60	0.00	15.19
		Others	36 (9)	0.62	0.00	0.00	0.04
		All	52 (10)	2.96 × 10^–3^	0.64	0.14	1.72
	C11S2804 (*OR5AN1*)	7	16 (1)	1.64 × 10^–4^	0.60	0.00	15.19
		Others	107 (24)	0.46	0.00	0.00	0.09
		All	123 (25)	0.024	0.64	0.40	0.87
	C11S3874 (*FIBP*)	7	16 (1)	1.64 × 10^–4^	1.08	0.09	15.19
		Others	0 (0)	NA	NA	NA	NA
		All	16 (1)	5.22 × 10^–7^	0.87	0.02	5.07
Q2	C21S1354 (*U2AF1*)	7	7 (1)	2.58 × 10^–4^	1.79	0.60	12.31
		Others	4 (1)	0.81	0.23	0.23	0.02
		All	11 (2)	6.56 × 10^–4^	1.07	0.52	1.99
	C2S4965 (*WDR75*)	7	13 (1)	3.51 × 10^–4^	1.22	0.04	15.25
		Others	0 (0)	NA	NA	NA	NA
		All	13 (1)	7.04 × 10^–5^	0.00	0.00	3.62
	C5S12 (*PLEKHG4B*)	7	24 (1)	3.80 × 10^–4^	0.71	0.00	12.92
		Others	4 (2)	0.75	0.00	0.00	0.00
		All	28 (3)	7.31 × 10^–4^	0.07	0.00	2.62
	C5S252 (*PLEKHG4B*)	7	24 (1)	3.80 × 10^–4^	0.71	0.00	12.92
		Others	4 (2)	0.75	0.00	0.00	0.00
		All	28 (3)	7.31 × 10^–4^	0.07	0.00	2.62
	C21S898 (*BRWD1*)	7	12 (2)	4.29 × 10^–4^	1.96	1.43	8.46
		Others	20 (4)	0.21	0.32	0.26	0.05
		All	32 (6)	9.69 × 10^–4^	1.30	0.85	0.99
	C21S1096 (*BRWD1*)	7	12 (2)	4.29 × 10^–4^	1.96	1.43	8.46
		Others	16 (3)	0.043	0.32	0.20	0.50
		All	28 (5)	8.84 × 10^–5^	1.30	0.80	1.85

The effect of a specific variant on the test of linkage (unadjusted LOD versus LOD adjusted for the variant) at the position of the gene is indicated as well as the proportion of the variance explained by the variant. Results are separated according to the set of families on which the analysis was restricted: in family 7 alone (7), in families other than family 7 (Others), and in all families combined (All). Causal SNPs and genes are in bold.

For trait Q1, C4S4935 (*VEGFC*) explained a highly significant proportion of the variance (33.1% in family 7, *p* = 7.9 × 10^–12^; 10.6% in all families combined, *p* = 1.1 × 10^–16^). This variant was unique to family 7; it entered the family through a single founder and was identified in 30 other family members. C6S2981 (*VEGFA*) was also significantly associated with trait Q1 (28.9% of the variance in family 7 was explained, *p* = 1.3 × 10^–7^; 11.9% of the variance in all families was explained, *p* = 9.9 × 10^–18^); it entered the family through a different founder and was found in 21 additional members (14 of whom were also carriers of the C4S4935 variant). Even though both variants shared a large number of carriers, each variant was still associated (at *p* < 10^–7^) when the other one was controlled for. C6S2981 was also identified and significantly associated in the other families, unlike the C6S2432 variant (which was in high linkage disequilibrium with C6S2981 in family 7); the possibility for C6S2432 (in *PSMB8*) to have a functional effect was thus excluded. The other variants from Table 3 that were associated with Q1 were also identified in the other families, but they showed no significant effect there, except for C11S3874 (*FIBP*), which was unique to family 7 (15.9% of the variance in family 7 was explained, *p* = 1.6 × 10^–4^; 5.0% of the variance in all families was explained, *p* = 5.2 × 10^–7^).

For trait Q2, most of the variants found to be associated in family 7 were found in other families; although these additional carriers did not improve the associations, their numbers were often too small to draw significant conclusions. C2S4965 (*WDR75*) was unique to family 7 and showed some level of association (15.2% of the variance in family 7 was explained, *p* = 3.5 × 10^–4^; 3.6% of the variance in all families was explained, *p* = 7.0 × 10^–5^).

## Discussion and conclusions

Our strategy of using an oligogenic segregation analysis as a prioritizing tool to identify specific rare functional variants that explain a significant proportion of the trait variance provides insights into efficient sequencing study design: By allocating sequencing resources to families that are more likely to harbor rare functional variants, costs can be reduced. This is especially important if the main focus of a study is the discovery of rare functional variants that are unlikely to segregate in more than a few families. Once a rare functional variant enters a family, it can segregate to many family members, increasing the likelihood of detecting its effect, so long as the variant entered the family a few generations ago; this warrants the use of large, multigenerational families in which many meioses occurred. Statistical validation and replication of the effects of rare variants will require an even larger sample size, without even the certainty of capturing the rare variants that are possibly unique to the families in which they were discovered; the importance of biological validation will be key in interpreting results.

Without prior knowledge of the GAW17 simulation answers and using an oligogenic segregation analysis as a prioritization tool for selecting the families most likely to harbor rare variants, we correctly identified two rare functional variants, *VEGFA* and *VEGFC*, including one private to family 7. Note that all genes with LOD scores greater than 4 in all families combined carried either a true susceptibility variant (C6S2981 for *VEGFA* and C4S4935 for *VEGFC*) or a variant in high linkage disequilibrium with a true causal one (C6S2432 for *PSMB8*). All other genes from Table 3 were false positives, and all had LOD scores below 2. The strength of linkage was thus indicative of the presence of true susceptibility genes.

In addition to being a successful strategy, our method is an efficient one with respect to reducing sequencing costs and to using resources conservatively by correctly eliminating traits unlikely to yield detectable results. For example, despite a heritability of 65%, Q4 was not under the influence of any of the genotyped exonic SNPs in the data set and was correctly eliminated based on our oligogenic segregation analysis, which showed that less than 14% of the total variance was due to a QTL. Although our strategy led to many fewer false positives than testing for all variants, other complementary design and/or analytic approaches will be needed to further decrease type I error. Because not all susceptibility variants for a given trait will be represented in family data, large samples of unrelated individuals will continue to provide a complementary design strategy.

## Competing interests

The authors declare that they have no competing interests.

## Authors’ contributions

FG and ML designed the study; FG, NMR and ML analyzed the data and interpreted the results; ML drafted the manuscript with contributions from FG and NMR. All authors read and approved the final manuscript.

## References

[B1] International HapMap Consortium A second generation human haplotype map of over 3.1 million SNPs Nature 2007 449 851 861 10.1038/nature06258 17943122 PMC2689609

[B2] Dickson SP Wang K Krantz I Hakonarson H Goldstein DB Rare variants create synthetic genome-wide associations PLoS Biol 2010 8 e1000294 10.1371/journal.pbio.1000294 20126254 PMC2811148

[B3] Li B Leal SM Methods for detecting associations with rare variants for common diseases: application to analysis of sequence data Am J Hum Genet 2008 83 311 321 10.1016/j.ajhg.2008.06.024 18691683 PMC2842185

[B4] Madsen BO Browning SR A groupwise association test for rare mutations using a weighted sum statistic PLoS Genet 2009 5 e1000384 10.1371/journal.pgen.1000384 19214210 PMC2633048

[B5] Manolio TA Collins FS Cox NJ Goldstein DB Hindorff LA Hunter DJ McCarthy MI Ramos EM Cardon LR Chakravarti A Finding the missing heritability of complex diseases Nature 2009 461 747 753 10.1038/nature08494 19812666 PMC2831613

[B6] Heath SC Markov chain Monte Carlo segregation and linkage analysis for oligogenic models Am J Hum Genet 1997 61 748 760 10.1086/515506 9326339 PMC1715966

[B7] Almasy L Blangero J Multipoint quantitative trait linkage analysis in general pedigrees Am J Hum Genet 1998 62 1198 1211 10.1086/301844 9545414 PMC1377101

